# CAR T cells and T cell engagers for autoimmunity—lessons from hematology

**DOI:** 10.1038/s41409-026-02808-1

**Published:** 2026-02-23

**Authors:** Sarah Kayser, Arnon Nagler

**Affiliations:** 1 Department of Hematology, Oncology and Cancer Immunology, Charité - Universitätsmedizin Berlin, corporate member of Freie Universität Berlin and Humboldt-Universität zu Berlin, Berlin, Germany; 2 Division of Hematology, Sheba Medical Center, Tel Hashomer, Israel

**Keywords:** Autoimmune diseases, Cancer immunotherapy

## Abstract

Autoimmune diseases are driven by dysregulated immune responses against self-antigens, resulting in chronic inflammation and progressive tissue damage. Chimeric antigen receptor (CAR) T cell therapy and T cell engagers, initially developed for the treatment of hematologic malignancies, have demonstrated that they can mediate profound and sustained depletion of pathogenic B cells, leading to long-term remissions in otherwise refractory diseases. In this review, we explore key insights gained from hematologic applications of CAR T cells and T cell engagers, and discuss how these lessons can guide the clinical use of these therapies in autoimmune diseases.

## Introduction

More than fifteen years have passed since the first chimeric antigen receptor (CAR) T cell was administered to a patient with hematologic malignancy [1–3]. Since then, CAR T-cell therapy has evolved from an experimental concept to an established, potentially curative treatment for refractory lymphoid leukemias, lymphomas, and multiple myeloma [4]. Multiple products targeting CD19 or B-cell maturation antigen (BCMA) have received regulatory approval, demonstrating that cellular therapies can incduce deep and durable clinical responses in otherwise incurable diseases.

Beyond these clinical successes, hematologic CAR T-cell therapy has generated a rich body of mechanistic, translational, and clinical insight. These lessons are highly relevant as cellular immunotherapy moves beyond oncology into immune-mediated diseases [5]. However, the therapeutic objectives differ fundamentally. In cancer, CAR T cells function primarily as cytotoxic effectors, and efficacy is often linked to robust expansion, persistence, and ongoing antigen engagement. In autoimmune diseases, by contrast, the goal is not continuous immune surveillance, but rather interruption of pathogenic immune circuits and restoration of durable self-tolerance.

A key question is whether the principles of CAR T cell therapy in malignancies translate to autoimmune disorders. This review discusses key lessons learned from hematologic CAR T-cell and T-cell engager therapies and explores their translational potential in autoimmune diseases.

## Lessons from hematology

### Rapid clinical adoption has been driven by early generation of high-level evidence

Over the past decade, CAR T-cell therapy has transformed the treatment landscape in hematologic malignancies. Between 2016 and 2023, more than 6,000 patients in the United States alone received CAR T-cell infusions, reflecting rapid clinical uptake [6]. In large B-cell lymphoma (LBCL), initial single-arm studies such as ZUMA-1 [7], JULIET [8], and TRANSCEND [9] demonstrated high complete remission rates in heavily pretreated patients, followed by randomized trials demonstrating superiority over standard chemoimmunotherapy in the second-line setting. These data firmly established CD19-directed CAR T-cell therapy as a standard of care for relapsed or refractory LBCL and support its earlier integration into treatment algorithms for selected high-risk patients. Ongoing clinical trials are now evaluating CAR T-cell therapy in frontline settings, underscoring a continued, evidence-driven shift toward earlier use. The same blueprint—robust prospective trials, standardized endpoints, and real-world validation—will be essential for advancing CAR T-cell therapy in autoimmune diseases.

### Patient selection is determined by biological fitness rather than age

Across hematologic malignancies, clinical trials and real-world data demonstrate that chronological age alone does not meaningfully limit the efficacy or safety of CAR T-cell therapy [10, 11]. When performance status and comorbidities are taken into account, outcomes in older adults mirror those of younger patients, whereas impaired functional status, elevated LDH, and high disease burden consistently predict inferior responses [12, 13]. Collectively, these data demonstrate that chronological age alone does not limit the efficacy or safety of CD19-directed CAR T-cell therapy in LBCL. These observations underscore that eligibility for cellular immunotherapy should be guided by biological rather than chronological fitness—a principle of particular relevance for autoimmune diseases, which frequently affect older populations.

### Timing and disease burden critically influence therapeutic success

Durable remissions are most often achieved in settings of lower tumor burden and earlier intervention, whereas high disease burden limits CAR T-cell expansion and increases relapse risk [14–17]. Prior treatment exposures similarly affect outcomes by depleting T-cell fitness [18] and altering antigen expression [19]. Notably, corticosteroid exposure used for CRS management does not appear to compromise CAR T-cell expansion, persistence, or efficacy [20, 21]. Translating these lessons to autoimmunity suggests that earlier use—before extensive tissue damage or broad immune dysregulation occurs—may enhance long-term benefit.

### CAR T-cell expansion and persistence

Intrinsic T-cell quality is a dominant determinant of efficacy. Superior outcomes correlate with leukapheresis material enriched for early memory T-cell subsets, preserved mitochondrial function, and low exhaustion signatures [22]. CAR construct design [23] and dosing strategies [24] further modulate efficacy and toxicity: altered signaling domains, binding kinetics, or fractionated infusions can achieve potent immune activity while limiting excessive activation. Lymphodepletion is also critical, as it provides space and homeostatic cytokine support for CAR T-cell expansion. Importantly, adequate fludarabine exposure has emerged as a major predictor of favorable outcomes [19, 25, 26]. Finally, the host immune environment—including baseline inflammation, cytokine profiles, and suppressive signals—shapes expansion, persistence, and relapse risk [27, 28]. Together, these lessons emphasize that optimal CAR T-cell responses depend on both product-intrinsic characteristics and host-related factors, a principle highly relevant for non-malignant indications.

### Predictive and adaptive strategies

Relapse remains common even after initial responses, highlighting the need for predictive and adaptive approaches. Dynamic biomarkers, such as CAR T-cell expansion kinetics, inflammatory signatures, antigen evolution, and circulating tumor DNA, can inform dose adjustments, timing of repeat infusions, or supportive interventions to maintain long-term disease control. Integrating these strategies into clinical decision-making offers a path toward personalized, durable therapy—both in oncology and potentially in autoimmunity.

Key mechanistic and clinical lessons from hematologic malignancies—and their potential translation to autoimmune disease—are summarized in Table 1.

**Table 1:** Translating lessons from hematologic malignancies to autoimmunity.

Lesson from hematology	Relevance or current status in autoimmunity
Antigen choice determines efficacy and escape (e.g., CD19 loss)	Partially defined. CD19 remains the main target; BCMA and dual-target CARs are under evaluation to address plasma-cell reservoirs and antigen escape.
Lymphodepletion enhances CAR T expansion and persistence	Uncertain. Most trials use fludarabine + cyclophosphamide, but the optimal intensity—or omission—in autoimmunity remains to be defined.
In vivo CAR T expansion correlates with response and toxicity	Under investigation. Expansion–efficacy thresholds and safety correlations are not yet established in autoimmune settings.
CRS and ICANS are predictable, manageable toxicities	Partly transferable. Reported mostly as low grade; systematic long-term safety data are still limited.
CAR T persistence supports durable remission	Unknown. Durable, drug-free remissions occur despite transient CAR detection; mechanisms of sustained tolerance require clarification.
T-cell fitness predicts outcome	Likely relevant. Prior immunomodulation and T-cell exhaustion may affect manufacturing success and efficacy.
Prior therapies influence product quality and target biology	Relevant. Previous B-cell–depleting or cytotoxic therapies can alter leukapheresis yields and antigen expression; washout periods need standardization.
Dose and infusion strategy affect efficacy and toxicity	Open question. Optimal CAR T-cell dose, fractionation, and retreatment remain undefined for autoimmune indications.
Allogeneic or off-the-shelf CAR T products expand access but raise rejection concerns	Exploratory. Early trials show feasibility; long-term immune reconstitution and safety data are awaited.
Bispecific T-cell engagers can bridge or substitute for salvage or CAR T therapy	Emerging option. Bispecifics provide rapid, reversible T-cell redirection and may serve as bridge, salvage, or alternative therapy.
Antigen switching (alternate targets) can rescue post-CAR relapse	Proof-of-concept. BCMA-directed CAR T cells have induced remission after CD19 CAR T failure in isolated cases.
Tumor microenvironmental resistance limits efficacy	Unclear analogs. Tissue niches maintaining autoreactive clones need characterization in autoimmune diseases.
Biomarkers guide intervention and retreatment	Critical unmet need. Predictive and mechanistic biomarkers remain underdeveloped for autoimmune CAR T and bispecific approaches.

Insights established in oncology and their current or potential relevance to autoimmune indications. References supporting these concepts are cited in the main text.

## From hematologic malignancies to autoimmunity

### Immune ablation and reconstitution as a therapeutic principle

The rationale for cellular therapy in autoimmune diseases is builds on decades of hematologic experience with immune ablation followed by immune reconstitution [29]. Autologous hematopoietic stem cell transplantation provided early proof of concept that transient, profound immunoablation can induce long-term, drug-free remission in selected patients with severe, refractory autoimmune diseases [29–35]. By eradicating autoreactive lymphocytes and allowing de novo immune reconstitution, autologous hematopoietic stem cell transplantation effectively “resets” immune tolerance. Mechanistically, this process involves depletion of pathogenic memory T and B cells, regeneration of a naïve-biased lymphocyte repertoire, reversal of T-cell exhaustion, and remodeling of autoreactive immune networks. However, relapse risk and treatment-related toxicity have limited its broader applicability [36]. CAR T-cell therapy represents an evolution of this concept, offering targeted and tissue-penetrant immunoablation with the potential for reduced systemic toxicity. By selectively eliminating pathogenic immune cell populations—most prominently B cells and plasma cells—CAR T cells can access inflamed tissues and secondary lymphoid organs while avoiding prolonged global immunosuppression.

### Early clinical evidence across autoimmune diseases

Early-phase clinical studies across a range of autoimmune diseases—including systemic lupus erythematosus, inflammatory myopathies, systemic sclerosis, neuromyelitis optica spectrum disorder, multiple sclerosis, and myasthenia gravis—have demonstrated rapid clinical and serological responses following CAR T-cell therapy, often enabling complete discontinuation of immunosuppressive treatment [5, 37]. Toxicity profiles have generally been favorable, with predominantly low-grade cytokine release syndrome (CRS) and minimal neurotoxicity. Mechanistically, this more favorable toxicity profile likely reflects lower antigen burden, reduced baseline systemic inflammation, and absence of tumor-driven cytokine priming, resulting in attenuated early CAR T-cell activation and lower peak cytokine release. Although patient numbers remain small, the consistency of responses across diverse autoimmune phenotypes supports the concept of a disease-agnostic immune reset in autoimmune diseases.

### Durability through immune reprogramming rather than persistence

A consistent observation across autoimmune CAR T-cell studies is the durability of clinical remission despite limited CAR T-cell persistence [37]. Instead, early elimination of pathogenic immune hierarchies appears to permit qualitative immune reprogramming, characterized by naïve-biased immune reconstitution, disruption of autoreactive memory compartments, and restoration of more tolerogenic immune networks. These findings suggest that transient cellular interventions can induce lasting disease control by reshaping immune architecture.

### Expanding targets and modalities

Beyond CD19, additional targets are being explored to broaden and refine immune modulation. CAR T-cell strategies directed against BCMA, CD20, CD38, and CD4 aim to extend depletion to plasma cells or pathogenic T-cell subsets, while chimeric autoantibody receptor (CAAR) T cells offer antigen-specific elimination of autoreactive B cells. These approaches seek to balance depth of immune depletion with preservation of protective immunity. Complementary antibody-based strategies, including CD38-directed antibodies [38] (e.g., daratumumab) and BCMA–CD3 bispecific T-cell engagers [39] (e.g., teclistamab), further support that pharmacologic plasma cell depletion and T-cell redirection can recapitulate key aspects of CAR T-cell–mediated immune modulation, while offering reversible, bridging, or post–CAR T therapeutic options.

## Clinical management

### Patient selection and pretreatment considerations

Recent expert-based recommendations from the European Society for Blood and Marrow Transplantation (EBMT) provide an emerging framework for the clinical use of CAR T-cell therapy in autoimmune diseases [5]. Patient selection principles largely mirror those established in hematologic malignancies, with disease-specific considerations for chronic organ damage and immune status. Chronological age alone should not preclude eligibility. Where feasible, background immunosuppressive therapy should be tapered before CAR T-cell infusion, although low-dose corticosteroids are permitted. Anti–T-cell–directed antibodies should be discontinued at least six weeks prior to leukapheresis or infusion to avoid impaired CAR T-cell expansion.

### Lymphodepletion and conditioning

Lymphodepletion with fludarabine and cyclophosphamide remains standard in current protocols. In non-malignant indications, however, its long-term consequences—including potential reproductive toxicity—require particular attention and patient counseling, especially in younger individuals. As in oncology, effective lymphodepletion is critical to enable CAR T-cell expansion and function, but the risk–benefit balance may differ in autoimmune disease.

### Toxicity profile and supportive care

Available clinical data indicate that patients treated for autoimmune diseases experience lower incidence and severity of CRS and Immune Effector Cell-Associated Neurotoxicity Syndrom (ICANS) compared with patients with hematologic malignancies [37]. Central nervous system involvement does not appear to confer an increased risk of neurotoxicity. Prophylactic granulocyte colony–stimulating factor is discouraged because of the potential risk of autoimmune disease flare. While prolonged cytopenias are a recognized complication in oncology, they are uncommon in autoimmune CAR T-cell cohorts, and the HEMATOTOX score—widely used in hematologic malignancies—has not been validated in this setting. Standard practice may include antibacterial, antiviral, anti–Pneumocystis, and antifungal prophylaxis during periods of neutropenia or lymphopenia. Long-term surveillance should also consider the potential risk of secondary malignancies, particularly in younger patients receiving lymphodepleting chemotherapy, although this has not yet been systematically observed in autoimmune cohorts. Vaccinations should be scheduled cautiously after immune reconstitution, as immune activation may theoretically precipitate disease recurrence. Given the complexity of immune monitoring, toxicity management, and disease assessment, the EBMT strongly recommends that CAR T-cell therapy for autoimmune diseases be performed exclusively at experienced centers with close collaboration between hematologists and autoimmune disease specialists. Joint, structured follow-up for at least six months is advised to ensure coordinated management of treatment-related toxicity, immune reconstitution, and disease activity.

## Conclusions and future directions

CAR T-cell therapy has produced unprecedented clinical response in refractory autoimmune diseases, inducing complete remission, elimination of pathogenic autoantibodies, and reversal of tissue injury with minimal adverse events. CD19-directed CAR T cells effectively deplete B cells and plasmablasts, and clinical benefit often persists after B-cell recovery and disappearance of CAR T cells, while protective immunity against bacterial and viral pathogens remains largely preserved. Whether CD19-mediated depletion alone is sufficient to establish durable immune tolerance—or whether additional compartments, such as CD19-negative plasma cells or autoreactive T cells, should also be targeted—remains unresolved. Antigen escape, well described in oncology, may emerge in autoimmunity, underscoring the potential need for alternative or additional targets such as BCMA.

Key clinical challenges include optimizing CAR target selection, dosing, and lymphodepletion regimens, as well as defining the sequence and positioning of CAR T-cell therapy and T-cell engagers within autoimmune treatment algorithms. Long-term safety considerations—including persistent immunodeficiency and the risk of secondary malignancies—require further systematic study. Practical limitations, such as high cost, limited global availability, and the requirement for specialized treatment centers, further constrain scalability and accessibility.

Future progress is likely to stem from next-generation approaches, including allogeneic “off-the-shelf” CARs, dual-specific or regulatory CAR T cells designed to promote immune tolerance, autologous RNA CAR T cells, emerging antigen targets, and complementary strategies such as monoclonal antibodies or bispecific T-cell engagers. Carefully designed prospective trials with extended follow-up are essential to define the therapeutic potential, durability, and long-term safety of these interventions, and to clarify their ultimate role in reshaping standards of care for autoimmune diseases. Major unresolved challenges and research priorities are summarized in Table 2.

**Table 2:** Open questions and future directions for CAR T-cell and T-cell engager therapy in autoimmunity.

Open question	Future direction
Target selection—Which antigens (CD19, BCMA, CD20, CD38, dual-target) achieve optimal depletion of autoreactive B cells while preserving protective immunity?	Comparative studies of antigen targets and dual-epitope strategies to prevent immune escape and relapse.
Mechanisms of immune reset—What molecular and cellular programs sustain long-term remission after transient CAR T persistence?	Longitudinal immune profiling to identify signatures of durable tolerance and relapse prediction.
Conditioning and dosing—Is lymphodepletion required, and what is the optimal dose or schedule in autoimmunity?	Controlled trials of reduced-intensity or omitted conditioning and repeat low-dose regimens.
In vivo CAR T-cell generation—Can lipid-nanoparticle or viral-vector delivery achieve immune reset with minimal toxicity?	Early-phase testing of in vivo CAR platforms and optimization of dosing, targeting, and durability.
Allogeneic and off-the-shelf CAR T cells—Are universal donor products safe without graft-versus-host risk?	Clinical assessment of engineered allogeneic CARs with immune-evasion and persistence controls.
Bispecific T-cell engagers—How durable are responses, and can bispecifics serve after CAR T failure?	Long-term bispecific follow-up and exploration of sequential or combination approaches with CAR T therapy.
CAR design and function—Can next-generation constructs (armoured, dual-specific, regulatory) promote tolerance over cytotoxicity?	Preclinical and translational studies of engineered CARs with regulatory or tolerogenic activity.
Safety and toxicity—What are the long-term risks of infection, immune dysregulation, or secondary malignancy?	Establishment of registries and standardized pharmacovigilance for autoimmune indications.
Long-term immunodeficiency—What are the risks of prolonged B- or T-cell depletion in autoimmune patients?	Longitudinal immune monitoring, infection risk assessment, and strategies to mitigate persistent immunodeficiency.
Antigen escape—Can pathogenic clones evade CAR T or T-cell engager therapy?	Sequential or multi-target approaches, dual-antigen CARs, and monitoring for escape variants.
Cost and accessibility—How can these therapies be made widely available?	Health-economic analyses, comparative trials with standard-of-care, and exploration of scalable manufacturing.
Clinical integration—How should CAR T therapy be positioned within treatment algorithms and disease stages?	Comparative trials versus biologics and B-cell-depleting agents; health-economic modeling.
Biomarkers and monitoring—Which biomarkers predict response, durability, and relapse?	Integration of single-cell and spatial multi-omics to identify predictive and mechanistic biomarkers.

Key unresolved issues, challenges, and opportunities. Supporting references are provided in the main text.
